# Evaluating Patient Preferences for Clinical Trial Endpoints in Early-Stage Cancer: A Discrete Choice Experiment in Canada

**DOI:** 10.3390/curroncol33060308

**Published:** 2026-05-26

**Authors:** Alexis T. Mickle, Frances Simbulan, Bianca Li, Kristoph Klein-Panneton, Conor L. Morrison, Sarah Walker, Karissa M. Johnston, Stephanie Snow

**Affiliations:** 1 Broadstreet HEOR, 177 W 7th Ave, Vancouver, BC V5Y 1L8, Canada; 2 AstraZeneca Canada Inc., 1004 Middlegate Rd, Mississauga, ON L4Y 1M4, Canada; 3 QEII Health Sciences Centre, Dalhousie University, 1796 Summer St, Halifax, NS B3H 3A7, Canada

**Keywords:** early-stage cancer, overall survival, clinical trial endpoints, patient preference, discrete choice experiment, health care surveys, patient preference, surrogate endpoint, disease-free survival, progression-free survival

## Abstract

To quantify preferences and trade-offs for non-overall survival (OS) endpoints among Canadian adults treated for early-stage cancers, participants completed ten choice sets in a discrete choice experiment. Standard-of-care (SoC) was compared to new treatment profiles defined by five attributes: five-year OS, two-year disease advancement, pathological complete response (pCR), and short- and long-term side effects. SoC attribute levels remained constant across respondents, except OS, which varied. For the new treatment, all attributes except OS (fixed as unknown) varied. Data were analyzed using logistic regression and presented as odds ratios (ORs) with 95% confidence intervals (CIs). Among 103 adults treated for early-stage gastrointestinal (*n* = 40), breast (*n* = 32), or lung (*n* = 31) cancer, median age was 59 (Q1, Q3: 43, 75) years; 46.6% were female. Each 25% decrease in SoC OS was associated with higher odds of choosing the new treatment (OR 3.49; 95% CI 2.82–4.31; *p* < 0.01). Reductions in disease advancement (OR 1.55; 1.26–1.91; *p* < 0.01) and mild or no short-term side effects versus severe (OR 6.67; 4.35–10.00) significantly increased selection odds; long-term side effects and pCR showed modest, non-significant influence. When OS data were available for SoC, OS strongly influenced decisions; without OS data for new treatments, participants prioritized disease control and short-term tolerability.

## Simple Summary

This survey assessed preferences among Canadian adults with early-stage cancers regarding cancer treatment evaluation, including endpoints beyond overall survival (OS), to better understand patient priorities in treatment decisions. Participants reviewed two hypothetical treatments and selected their preferred option based on five-year OS, two-year risk of cancer advancement, the likelihood of the treatment eliminating detectable cancer in tissue, and short- and long-term side effects. Among 103 respondents, treatments with higher OS, lower risk of cancer progression, which can affect quality of life and future treatment options, and reduced short-term or any long-term side effects were preferred. In supplemental questions, nearly half of participants supported access to a new treatment that lowered cancer progression, even if long-term survival benefits were not yet known. These findings highlight that, while OS remains highly influential in treatment decisions, people also prioritize delaying cancer progression and minimizing side effects when considering new therapies.

## 1. Introduction

Cancer remains a leading cause of death in Canada and globally [1,2,3]. For individuals with early-stage cancers, treatment pathways are guided by clinical risk evaluation, including cancer type, tumour stage, and biomarker status. The goal is to achieve cure and prevent recurrence. Surgery is performed where possible, and treatment may include neoadjuvant or adjuvant therapies [4,5,6,7]. Improvements in curative surgical outcomes and reduced risk of recurrence may be associated with increased overall survival (OS) and improved health-related quality of life (HRQoL) [8].

In clinical trials, efficacy has traditionally been assessed using OS, defined as “the time from randomization until death from any cause” [9]. OS is well understood among clinicians and decision-makers and represents a key outcome for advanced or metastatic cancers. However, there are challenges in using OS as a primary endpoint for early-stage cancers, prompting discussion of alternative endpoints that may better reflect curative intent, including a cancer-free state and reducing recurrence risk [9,10].

Clinical trials in early-stage cancers often use earlier endpoints (non-OS endpoints), which provide an early indication of treatment efficacy, allowing comparison of treatments before long-term OS data are available. For example, pathological complete response (pCR) assesses the presence of residual cancer cells in resected tissue following neoadjuvant therapy and surgery [11]. While pCR is commonly used as an early endpoint in some early-stage cancer settings, its relationship with long-term outcomes such as OS is not established across cancer types and treatment contexts. However, evidence linking pCR with improved long-term survival has been demonstrated in breast cancer [12,13] and, to a lesser extent, NSCLC [14]. Time-based measures, such as event-free survival (EFS), recurrence-free survival (RFS), and disease-free survival (DFS), evaluate the duration from treatment to the occurrence of clinical events or disease recurrence [15,16].

Assessment of OS in clinical trials presents both practical and methodological challenges for treatments with curative intent, particularly because long follow-up is required [17,18] and OS can be confounded by subsequent treatments [18,19]. Reliance solely on OS may delay timely access to new therapies, as decision makers prefer to wait for prolonged clinical trials to reach maturity before approving new options for patients [20]. Non-OS endpoints may provide an earlier indication of efficacy, potentially providing early access [21]. There is also growing recognition of the importance of connecting patients’ values with clinical trial endpoints throughout all stages of cancer, including the value placed on treatment impacts on HRQoL, symptom reduction, and maintenance of physical functioning, which non-OS endpoints may better capture [11,19,21]. In this context, clinical trial endpoints relate to outcomes that are meaningful to individuals, where survival reflects longevity, disease advancement reflects the risk of recurrence and ongoing disease burden, and treatment-related side effects reflect impacts on daily functioning and quality of life.

This research group previously conducted a qualitative study examining the importance of non-OS endpoints among people treated for early-stage cancers [22]. This research found that individuals with early-stage cancers valued non-OS endpoints because they align with treatment goals and provide information on disease progression, treatment-related side effects, and HRQoL. While quantitative studies have examined preferences for non-OS endpoints in advanced-stage cancers [23,24], less is known about preferences for endpoints used in trials for early-stage disease or when OS data are not yet available.

The objective of this study was to estimate the relative importance individuals treated for early-stage GI, lung, or breast cancers place on clinical trial endpoints and risk of side effects and the trade-offs they are willing to make between these outcomes when making hypothetical treatment decisions.

## 2. Materials and Methods

The discrete choice experiment (DCE) was conducted in Canada between April and May 2025 to estimate the preferences of people diagnosed with early-stage cancer, defined as cancer that had not metastasized beyond local lymph nodes and was treated with curative intent.

Participants were adults residing in Canada, fluent in English or French, and receiving or having previously received treatment for early-stage GI, breast, or lung cancer. To ensure adequate representation of key influential factors anticipated *a priori*, recruitment targets were set for cancer types and sociodemographic characteristics, including race/ethnicity, geography, education and household income (Table S1).

### 2.1. Study Design

Participants were first provided introductory materials that explained clinical trials, common clinical trial endpoints, the role and relevance of non-OS endpoints (e.g., disease advancement and pCR) in cancer treatment evaluation and approval, and short- and long-term side effects, followed by instructions to complete the DCE (Figure S1). The introductory materials included detailed, lay-language explanations supported by illustrative graphics. Definitions for key terms, including OS, disease advancement, pCR, and short- and long-term side effects, were accessible throughout the survey via pop-up or hover text. A set of comprehension questions was then administered to reinforce understanding of key concepts; incorrect responses triggered the display of the relevant explanatory text before participants proceeded to the DCE (Figure S2). Following the DCE, participants responded to clinical and demographic questions, along with items designed to capture their perspectives on the approval process for new cancer therapies (Figure S3). All questions were presented as drop-down lists or radio buttons, with free-text responses not permitted. All survey materials were available in both French and English, allowing participants to complete the survey in their preferred language.

#### DCE

**Figure 1:**
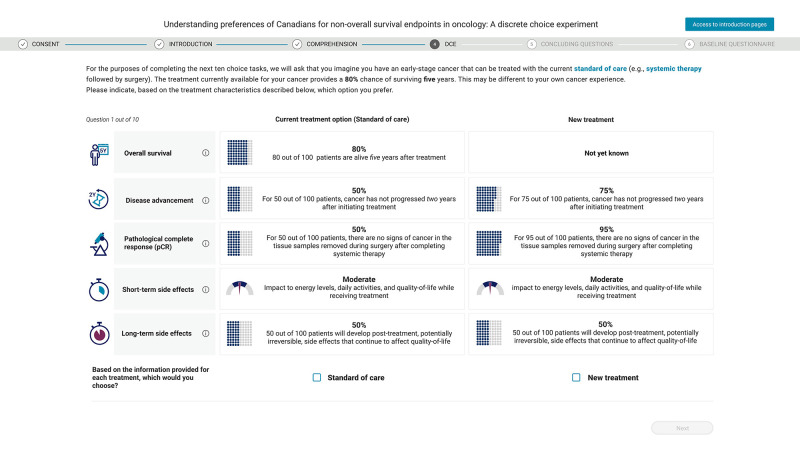
Sample discrete choice experiment choice task.

The selection of DCE attributes and levels was iterative and informed by a targeted literature review [23,25,26], qualitative research [22], and clinical expert consultation. The initial list for consideration included EFS, RFS, and DFS; however, as they have overlapping definitions [9,15,16] and capture related aspects of disease recurrence or progression, there was potential for correlation between these attributes. Including these endpoints as separate attributes would introduce structural challenges in the DCE design, as they cannot be varied independently without generating clinically implausible combinations, while forcing them to vary together would limit the variation needed to estimate preferences. In addition, the nuanced distinctions between these endpoints may be difficult for respondents to interpret and meaningfully trade off, increasing cognitive burden. To address these considerations, the endpoints were consolidated into a single composite attribute, disease advancement, resulting in a concept that is more interpretable and meaningful for respondents. As HRQoL is closely related to side effects [22,27], the potential impact was incorporated into the descriptions of side effects. All survey materials (protocol, recruitment criteria, survey introduction explanations, DCE attributes and levels, and clinical and demographic questionnaires), analyses, and results were reviewed by a Canadian clinical oncology physician (author S. Snow) to ensure clinical accuracy, relevance, and alignment with real-world practice. The final included attributes were five-year OS, two-year disease advancement, pCR, and risk of short- and long-term side effects (Table 1; Figure 1).

Clinically relevant attribute values were informed by published survival estimates from trials of existing therapies for the cancer types under study [28,29,30,31]. Expected values for non-OS endpoints associated with the new treatment with unknown OS represented competitive alternatives to the SoC treatment with known OS.

An orthogonal fractional factorial DCE design was generated using the Hahn and Shapiro catalogue (plan code 17a; masterplan 3; columns 1, 2, 3, 4) [32] and included 9 choice tasks (one block). The design was optimized to estimate main effects only, under the assumption of no interactions between attributes. No *a priori* knowledge of signs or true parameters was incorporated.

The DCE included two treatment profiles in each choice task: a SoC profile and a new treatment profile, with choices labelled as such (Figure 1). For the SoC profile, values for two-year disease advancement, pCR, short-term side effects, and long-term side effects were fixed; three different values were used for five-year OS, which was fixed at the respondent level such that each participant was exposed to a single OS value across all choice tasks but varied across participants. In the new treatment profile, five-year OS was fixed at ‘not yet known’. Values for disease advancement, pCR, and short- and long-term side effects were set to be equivalent to or better than those in the SoC profile to align with real-world scenarios. Both the choice task order and attribute order were randomized across participants but not randomized within a respondent’s questionnaire. All attributes were presented in every choice task (full-profile design). Responses were forced choice, with no opt-out option to reflect a scenario in which individuals with early-stage cancers would be expected to consider active treatment, thereby ensuring engagement with trade-offs across treatment attributes. No impossible profiles were generated (e.g., a treatment administered orally but associated with injection-site reactions); therefore, no adjustments to the choice sets were required. Choice sets were evaluated for orthogonality, level balance, minimal overlap, and utility balance. The experimental design included two logic tests: one choice task was dominant for the SoC treatment; one choice was repeated to check consistency.

### 2.2. Sample Size

Sample size was guided by established recommendations for DCEs and feasibility considerations [33]. Using the rule of thumb, N ≥ T (K/S), where T is the number of choice tasks per respondent, K is the maximum number of attribute levels, and S is the number of alternatives, the minimum required sample size was 83. Based on this estimate, a target sample of 100 participants was considered sufficient to estimate main effects with acceptable precision.

### 2.3. Recruitment

Screening, recruitment, and moderation were conducted by Qualtrics [34]. Eligibility was assessed using an online screening questionnaire aligned with inclusion criteria and sampling targets (Table S1). Sampling quotas were established a priori to ensure broad representation across key clinical and demographic characteristics, including cancer type, age, sex, race/ethnicity, geographic location of residence (rural/urban), socio-economic status, time since diagnosis, and recurrence experience, and to allow for exploratory subgroup analyses. Eligible individuals completed a moderated online questionnaire featuring the discrete choice sets, with moderators guiding participants and verifying response accuracy and authenticity. Participation was voluntary, with a $70 CAD honorarium provided upon completion.

### 2.4. Questionnaire Hosting and Deployment

The moderated questionnaire was administered using a customized online platform developed in-house; all data collected were stored on secure Egnyte servers [35]. A pilot of the DCE was conducted, resulting in feedback related to clarity of wording, comprehensibility of explanations, ease of completing the choice tasks, and survey flow and functionality. Refinements were implemented prior to administration in the target population.

This study was approved by the WCG ethics review board on 25 October 2024 (IRB tracking #_20244221) [36] and adhered to the Discrete Choice Experiment Reporting Checklist (DIRECT) [37].

### 2.5. Analysis

Participant demographic and clinical characteristics are reported as counts with proportions and/or as means with standard deviation (SD) or medians with interquartile range (IQR). Data obtained were analyzed using multivariable logistic regression models in *R* (R version 4.4.1) [38] and are presented as odds ratios (ORs) with 95% confidence intervals (CIs) and *p*-values, with *p* < 0.05 considered statistically significant. A standard logistic regression model was used to estimate population-level (aggregate) preferences for treatment attributes. Consistent with the study objective and sample size, more complex model specifications (e.g., mixed logit models) were not pursued. Logistic regression was chosen for interpretability and suitability for choice-based data, given the minimal within-choice variation; however, between-participant variation was not modelled. All choice task responses, including those from logic (dominance and consistency) questions, were included in the full analysis set. All questions were mandatory for progression and submission, preventing any missing data. No responses were removed from the final analysis set.

Levels for OS for the SoC treatment were initially set at 25%, 50%, and 90%. Interim review after 30 surveys indicated near-deterministic choices at the extreme values (new treatment at 25% OS, SoC at 90% OS), with indifference observed at 50%. To improve discriminatory power, OS levels were revised to 40%, 60%, and 80%. Although the small number of respondents per OS value (e.g., 12 at 50%) limits assessment of non-linearity, observed trends were considered sufficiently linear for OS to be modelled as a continuous variable.

Results were also expressed as marginal rates of substitution between the five-year OS coefficient and the other attribute coefficients to estimate trade-offs that participants were willing to make between OS and a specified change in the other attributes.

Stratified analyses were conducted based on demographic and clinical characteristics identified *a priori* as potential drivers of preference for oncology endpoints based on findings from a previously conducted qualitative study [39,40,41]. Stratifying variables included cancer type, age at diagnosis, time since diagnosis, recurrence history, and side effects experienced during treatment. Stratification was also conducted by participants’ response (yes/no) to an exploratory post-DCE question regarding whether new treatments should be made available based on alternative endpoint data in the absence of OS data. Comparisons of preferences across the three cancer types were evaluated using the smallest Bonferroni-corrected z-test *p*-values, while comparisons for all other (dichotomous) variables were conducted using z-tests. Statistical significance of between-group differences was defined as *p* < 0.05.

## 3. Results

A total of 106 participants initiated the survey, of whom 103 completed it, corresponding to a completion rate of 97%. The median survey completion time was 18 min, 24 s (range: 8–46 min).

### 3.1. Participant Clinical and Demographic Characteristics

A total of 103 adults diagnosed with early-stage cancer participated in the DCE, including those treated for early-stage GI cancer (*n* = 40, 38.8%), breast cancer (*n* = 32, 31.1%), and lung cancer (*n* = 31, 30.1%). Among participants with GI cancers, 12 (11.7%) had gastric cancer; 11 (10.7%) had hepatocellular carcinoma; and nine (8.7%) had esophageal cancer. The median age (IQR) was 59 (43, 75) years, and 46.6% were female (Table 2). Most participants reported that they were diagnosed at stage I (GI, 55.0%; non-small cell lung cancer (NSCLC), 75.0%; breast cancer, 81.2%), although the stage at diagnosis ranged from stage 0 (GI, NSCLC, and ductal carcinoma in situ for breast cancer) to stage A (liver cancer) or stage II (breast, other GI, and NSCLC). While most lung cancer diagnoses were for NSCLC, small cell lung cancer was reported among 22.6% of those with lung cancer. All participants indicated they had received surgery, often in combination with neoadjuvant or adjuvant treatments, including chemotherapy (*n* = 37, 35.9%), chemoradiotherapy (*n* = 55, 53.4%), radiation (*n* = 16, 15.5%), or ablation (*n* = 9, 22.5%) for participants with GI cancer. Most participants had completed treatment at the time of the study, although 43 (41.7%) were currently undergoing treatment for their primary cancer diagnosis or developed a recurrence. Additional clinical and demographic characteristics stratified by cancer type are presented in the Supplementary Materials (GI, Table S2; lung, Table S3; breast, Table S4).

### 3.2. DCE Results

Dominance and consistency testing suggested good participant engagement, with 85.4% (*n* = 88) passing both checks; 7.8% (*n* = 8) failed dominance only, 7.8% (*n* = 8) failed consistency only, and 1.0% (*n* = 1) failed both. A sensitivity analysis excluding individuals who failed either check yielded similar results; therefore, all survey responses were included in the full analysis set (FAS).

**Figure 2:**
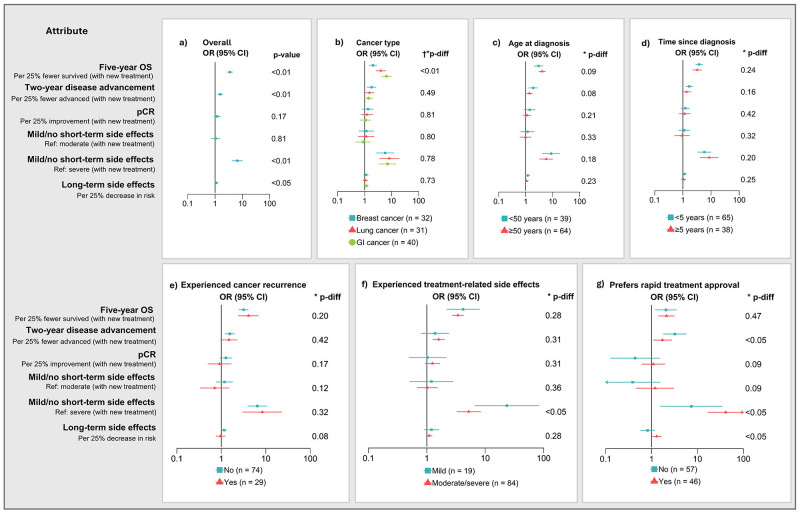
Discrete choice experiment results (**a**) overall, and stratified by participant clinical and demographic characteristics, including (**b**) cancer type, (**c**) age, (**d**) time since diagnosis, (**e**) experienced cancer recurrence, (**f**) experienced treatment-related side effects, and (**g**) prefers rapid treatment approval. Abbreviations: CI, confidence interval; GI, gastrointestinal; OR, odds ratio; OS, overall survival; pCR, pathological complete response. Notes: * *p*-values are from unpaired z-tests for two groups or the smallest Bonferroni-corrected z-test *p*-value when more than two groups are compared; † Bonferroni-corrected *p*-values for five-year OS are: lung versus gastrointestinal cancer, *p* = 0.18; lung versus breast cancer, *p* = 0.02; breast versus gastrointestinal cancer, *p* < 0.01.

Preferences are presented as the odds that a participant would choose the new treatment with no OS data available, based on five-year OS of the SoC, two-year disease advancement (i.e., non-OS endpoints EFS, DFS, RFS), rate of pCR, and risks of short- and long-term side effects (Figure 2a; Table S5). Participants were 3.5 times more likely to select the new treatment (OR = 3.49; 95% CI 2.82–4.31; *p* < 0.01) for every 25% decrease in the SoC five-year OS. For every 25% improvement in disease advancement two years after starting a new treatment, individuals were 1.55 times more likely to select that treatment (OR = 1.55; 95% CI 1.26–1.91; *p* < 0.01). Participants were 6.7 times more likely to choose a treatment with no or mild side effects compared to a new treatment with severe short-term side effects (OR = 6.67; 95% CI 4.35–10.00; *p* < 0.01). For every 25% decrease in the risk of long-term side effects, participants were 1.11 times more likely to choose the treatment (OR = 1.11; 95% CI 1.00–1.23; *p* < 0.05).

Preferences favoured the new treatment with a 25% improvement in pCR, showing an increase in odds (OR = 1.20; 95% CI 0.92–1.5; *p* = 0.17), although it was not statistically significant. Similarly, preferences for a new treatment with mild/no versus moderate short-term side effects were not statistically significant (OR = 1.04; 95% CI 0.73–1.49; *p* = 0.81).

#### 3.2.1. Stratified Analyses

Preference for the new treatment increased as five-year OS for the SoC worsened, despite the lack of data on five-year OS for the new treatment. This effect was most pronounced among participants treated for gastrointestinal cancer (OR = 6.45; 95% CI 9.79–4.22; *p* < 0.01), followed by those with lung cancer (OR = 4.02; 95% CI 6.08–2.66; *p* < 0.01), and was lowest among those with breast cancer (OR = 2.05; 95% CI 2.80–1.49; *p* < 0.01) for a 25% decrease in OS. Differences in preference between participants with lung vs. breast (*p*-diff = 0.02) and breast vs. GI (*p*-diff < 0.01) cancers were statistically significant, whereas the difference between lung and GI cancers was not (*p*-diff = 0.18; Figure 2b). No statistically significant differences in preference were observed based on age at diagnosis, time at diagnosis, or experience with cancer recurrence (Figure 2c–e).

Individuals who had experienced only mild side effects and those who had experienced moderate or severe side effects were both significantly more likely to select a treatment with mild/no short-term side effects vs. one with severe side effects (experienced mild: OR = 23.48; 95% CI 6.64–83.02; *p* < 0.01; experienced moderate/severe: OR = 5.20; 95% CI 3.28–8.25; *p* < 0.01). However, the aversion was greater among those whose prior side effects were mild, demonstrating a statistically significant difference between groups (*p*-diff < 0.05; Figure 2f).

All participants preferred treatments with greater improvements in two-year disease advancement. In exploratory questions following the DCE, participants who supported early approval of new treatments based on non-OS endpoints showed significant preference for this attribute (OR = 1.73; 95% CI 1.10–2.72; *p* < 0.05; Table 3). Additionally, statistically significant differences were observed for side-effect attributes. Participants who supported early approval based on non-OS endpoints were more likely to select a treatment with no/mild short-term side effects (OR = 41.24; 95% CI 17.0–100.1; *p* < 0.01) relative to those who did not support early approval (OR = 7.34; 95% CI 1.58–34.1; *p* < 0.05; *p*-diff < 0.05). They also preferred a treatment with a 25% lower risk of long-term side effects (OR = 1.29; 95% CI 1.03–1.63; *p* < 0.05) compared with those not supporting early approval (OR = 0.82; 95% CI 0.58–1.17; *p* = 0.28; *p*-diff < 0.05; Figure 2g).

#### 3.2.2. Point of Indifference/Willingness to Trade for OS

Participants were willing to accept reductions in established five-year OS in exchange for improvements in non-OS treatment attributes in a novel therapy where OS benefit had not yet been demonstrated. The largest trade-off was observed for avoiding short-term side effects (38.4%), followed by reducing disease advancement (8.9%), improving pCR (3.7%), reducing long-term side effects (2.1%), and avoiding moderate short-term side effects (0.8%; Table S6).

## 4. Discussion

Participants demonstrated stronger preferences for treatments not only with improved five-year OS but also for those offering reductions in disease advancement and side-effect burden, with these attributes being particularly influential when OS data for the new treatment were not yet available.

Preferences favoured the new treatment with improvements in pCR, although the results were not statistically significant. Given these results, measures of disease advancement and side effects are aligned with patient values for endpoints in clinical trials. Participants were willing to select a treatment in the absence of OS data, and nearly half of the participants supported access to new treatments showing clinical benefits on non-OS endpoints, even without mature OS data, indicating support for earlier access when meaningful improvements are shown.

Prior research in early-stage cancer demonstrates the value patients place on non-OS endpoints, particularly pCR and early measures of disease response, as personally meaningful and relatable to treatment experience [22,42]. For example, a 2016 study in early-stage breast cancer found pCR to be the most important outcome, ranking above DFS and OS [43]. A 2023 DCE in HER2-positive early-stage breast cancer similarly reported the highest preference for increases in pCR, followed by five-year DFS [27]. Pathologic complete response and time for cancer to respond to treatment were found to be particularly relevant for decision-making among people with breast cancer [27,41,43]. Participants in this DCE showed only a modest, non-statistically significant preference for higher pCR rates, which may reflect uncertainty in how this endpoint relates to longer-term outcomes such as OS across cancer types and treatment contexts, despite evidence supporting this association in some settings [12,14]. Clearer links between pCR improvements and meaningful long-term outcomes may enhance the perceived value. Overall, these studies reinforce the relevance of non-OS endpoints while illustrating variability in how they are prioritized by patients.

This study underscores the importance of maintaining HRQoL during treatment, reflected in a strong preference for treatments with reduced severe short-term side effects. Similar findings have been reported in previous studies among patients with NSCLC, where efficacy differences drove clinical improvements, but safety became the primary driver when efficacy was comparable [23,44]. Mild short-term side effects were comparatively less influential, consistent with prior reports emphasizing the prioritization of severe side effects and efficacy [27]. The perceived importance of safety also varied by type of side effect; for example, hair loss, although typically mild, was frequently prioritized for avoidance [23,43,45].

A qualitative study conducted as part of this research initiative in individuals with early-stage cancers found non-OS endpoints to be personally meaningful and distinct from OS, capturing aspects such as recurrence and HRQoL that survival alone could not [22]. These results are consistent with the DCE results, where attributes reflecting HRQoL (e.g., short-term side effects) and recurrence (e.g., disease advancement) were highly valued. While qualitative studies allow in-depth exploration, DCEs rely on participants’ pre-existing understanding, highlighting the need for patient education to support informed decision-making [46]. Broader education among patients, clinicians, and decision-makers is particularly important for early-stage cancer treatments, where precedent is limited compared with advanced disease trials [47]. Without clear communication of how non-OS endpoints are measured, their utility in decision-making may remain constrained [48,49,50].

Preferences were expressed as trade-offs in five-year OS, conceptually similar to a willingness to pay approach [51]. Willingness to trade OS in the SOC to avoid short-term side effects and reduce disease advancement highlights the importance of these attributes in treatment selection. Strong preferences for non-OS endpoints suggest that early access to treatments with meaningful clinical benefit and a manageable safety profile may be acceptable. Additionally, a willingness to trade small OS decrements for improvements in pCR or reductions in long-term side effects indicates that evidence linking these endpoints to meaningful long-term outcomes could enhance their perceived value.

Stratified analyses showed that the relative importance of five-year OS varied by cancer type. Individuals with breast cancer generally have more treatment options and a favourable prognosis, whereas those with lung or GI cancers have fewer options and poorer outcomes [28]. Preference for treatments improving OS was strongest among GI cancer participants, followed by lung and breast cancer, reflecting both prognoses and lived experience. Those at higher risk of poor outcomes may prioritize novel treatments offering improved HRQoL or reduced disease progression, even without mature OS data. Perceptions of current SoC also influence decisions; less effective or more burdensome therapies may increase the appeal of novel options.

Stability of preference reflects how treatment priorities may evolve through a person’s cancer experience [52]. Evidence shows preferences often shift based on diagnosis and treatment experiences. For instance, a 2020 survey of breast cancer patients found that those with more than six lines of therapy prioritized serious side effects and treatment burden more than those with only one line [41]. Similarly, participants who had experienced severe short-term side effects were less motivated to avoid them in this study, possibly reflecting greater confidence in managing adverse events or familiarity with outcomes. Preferences also vary by time since diagnosis, hormone receptor status, stage, and age. In a study in China, individuals with lung cancer placed less importance on OS than family members or oncologists, highlighting the importance of lived experiences [46]. Such considerations are important when interpreting preference studies beyond patient populations, including caregivers, physicians, and family members [39].

Participants’ support for earlier approval of therapies based on non-OS endpoint data was examined. Those favouring accelerated approval placed greater importance on minimizing short- and long-term side effect risks than participants who did not support earlier approval, while showing smaller preferences for reductions in two-year disease advancement. This suggests an emphasis on safety alongside timely access. Notably, participants’ views on what others should be able to choose did not always align with their own preferences, highlighting the distinction between personal and societal treatment choices.

A 2023 DCE conducted by Forrest et al. among older adults with personal or close experience with cancer found strong preferences for survival outcomes and minimizing US Food and Drug Administration (FDA) approval delays [39]. In this study, participants were willing to wait over a year for survival data when non-OS endpoints poorly predicted survival, leading the authors to conclude endpoints hold value primarily through their link to OS. The authors noted the FDA may underestimate individuals’ willingness to delay access for greater certainty of survival benefits. This study included a large sample including people with more cancer types and their caregivers, which may have influenced the relative importance placed on survival versus HRQoL [39].

Conventional HTA models are typically structured around survival outcomes such as OS and may not fully capture the value of other clinically meaningful outcomes, including reductions in disease activity and improvements in HRQoL. Along with growing recognition of the importance of non-OS endpoints in oncology clinical trials, regulatory bodies such as the FDA and European Medicines Agency (EMA) have both acknowledged the benefits of facilitating earlier access to treatments and expanded marketing authorization programs to allow rapid reviews of evidence-based non-OS endpoints from early-phase clinical trials when there is a correlation between the non-OS endpoint and OS [13,53,54,55]. Non-OS endpoints have also been used in traditional approval processes, although there must be stronger evidence supporting a relationship between the non-OS endpoints and OS [13,53,56]. This study demonstrates that the regulatory acceptance of non-OS endpoints is not simply a methodological convenience but is also aligned with patient values.

The recruitment strategy minimized bias and supported generalizable findings by targeting participants with direct experience of early-stage cancer treatments and using stratified sampling to ensure representation across key subgroups, including racial minorities, age ranges, treatment status, and relapse history. Concepts potentially influencing preferences, such as links between early non-OS endpoints and mature OS, were introduced only after the DCE. Additional strengths included independent moderators, descriptive introductory materials, and accessible materials to support comprehension and meaningful responses. The DCE attributes and levels were informed by qualitative interviews and targeted literature reviews, ensuring patient relevance and input from a Canadian medical oncologist enhanced clinical validity and interpretation.

This study’s sample size was sufficient for estimating main effects but limited the power of subgroup analyses, so these results should be interpreted cautiously. This study focused on estimating aggregate preferences for treatment attributes rather than characterizing preference heterogeneity. While more flexible model specifications (e.g., mixed logit or latent class models) can provide additional insight into heterogeneity, this study was not designed to support these analyses given the sample size and primary study objectives. A larger sample size would likely be required to support more complex modelling approaches and robust estimation of random effects. The analysis therefore assumed no interaction effects between attributes, a common and pragmatic approach in DCE studies of this size [57]. However, this may oversimplify real-world decision-making where preferences for one attribute depend on the levels of others. Volunteer participants, such as those recruited from a Qualtrics panel for this study, may be more engaged in their care or research and may differ from the broader population in terms of digital literacy and socioeconomic characteristics, introducing potential selection bias and affecting generalizability. Recruitment focused on individuals treated with a curative intent for breast, lung or GI cancers, which may limit applicability to more aggressive disease or other cancer types. Further research is needed to better understand how treatment preferences may differ across these characteristics. All demographic and clinical characteristics were self-reported, which may have led to inaccuracies; for example, participants reporting surgery for small cell lung cancer, a treatment that is uncommon for this cancer type, suggests possible reporting errors. Cognitive burden in completing the DCE and interpreting complex concepts, such as time-based non-OS endpoints, is a key limitation, as even common endpoints like OS or time to progression are not always well understood [41]. To mitigate this burden, the number of attributes was limited to the most relevant; a composite two-year disease advancement attribute was used; the SoC profile remained fixed; and attribute order was kept consistent across tasks. Plain language descriptions were provided and reviewed by a medical oncologist, and questions on non-OS endpoints for rapid approval were placed at the end to minimize influence on preferences and reduce fatigue. The absence of an opt-out option in the DCE may limit generalizability to scenarios in which individuals defer or refuse treatment; however, the forced-choice design was intentionally selected to evaluate trade-offs between treatment attributes among individuals considering active treatment, consistent with the study objectives. Finally, it should be noted that mid-study modification of OS levels (from 25–90% to 40–80%) may have introduced some inconsistency across respondents; however, as OS was fixed at the respondent level, each participant was exposed to only a single OS value across all choice tasks, and this change reflects a refinement of the range of values rather than within-respondent variation.

## 5. Conclusions

Despite the growing use of non-OS endpoints in early-stage oncology trials, uncertainty remains regarding their validity as surrogates for OS [18], and their acceptance by decision-makers, clinicians, and patients [19]. Evidence from this study indicates that individuals with early-stage cancers value treatment outcomes beyond survival, including disease advancement and both short- and long-term side effects. Importantly, these attributes influenced preferences even when OS data for a new treatment were not yet available, suggesting that individuals are willing to make treatment decisions under conditions of uncertainty. These findings support the relevance of non-OS endpoints in clinical trial design and regulatory decision-making, particularly in early-stage settings where survival data take years to mature. Interpretation of OS in early-stage oncology is further complicated by the increasing availability of effective subsequent lines of therapy and ongoing therapeutic innovation across tumour types [18,19]. Following completion of randomized treatment, oncologists and people with cancer may have access to a wide range of subsequent treatment options, which may confound or dilute survival differences attributable to earlier interventions. In this context, reliance solely on mature OS data may delay access to therapies that offer clinically meaningful benefits aligned with patient priorities. While preferences did not vary substantially across most subgroups in the current study, there was some evidence that disease experience influenced the relative importance of treatment attributes. Greater emphasis on communicating the meaning and implications of non-OS endpoints may further support informed, patient-centred decision-making and improve understanding.

Emerging evidence underscores the role of non-OS endpoints in treatment decision-making and the value that this would hold for individuals, but further clinical research is needed. Future studies could examine factors driving treatment preferences, including risk aversion and how clinicians communicate the relevance of treatment endpoints, to better support shared decision-making and reduce uncertainty.

## Figures and Tables

**Table 1:** Discrete choice experiment—attributes and levels.

Attribute & Description	Levels	
	Standard-of-Care (Fixed)	New Treatment (Varies)
**Overall survival** (fixed at the respondent level) Attribute description: *The proportion of people who are diagnosed with cancer and are alive, with or without cancer, five years after starting treatment* DCE attribute text: *X out of 100 (xx%) patients are alive five years after treatment* (standard-of-care); *not yet known*	25% 40% * 50% 60% * 80% * 90%	Not yet known
**Disease advancement** Attribute description: *Among a group of people who are diagnosed with cancer and undergoing a similar treatment (e.g., those enrolled in a clinical trial), the percentage who have no evidence of disease advancement (DFS, RFS, or EFS) over a given period (e.g., two years) after starting treatment* DCE attribute text: *For X out of 100 (xx%) patients, cancer has not progressed two years after treatment*	50%	50% 75% 95%
**Pathological complete response (pCR)** Attribute description: *Pathological complete response, or pCR, is achieved when there are no surviving cancer cells in a person’s tissue samples removed with surgery. Tissue samples are removed during surgery and are examined under a microscope to see if the treatment was successful* DCE attribute text: *For X out of 100 (xx%) patients, there are no signs of cancer in the tissue samples removed during surgery or biopsy after completing systemic therapy*	50%	50% 75% 95%
**Short-term side effects** Attribute description: *Short-term side effects occur during treatment and go away once treatment is complete. These side effects may include hair loss during chemotherapy, nausea, or vomiting. These side effects can impact energy levels, daily activities, and quality of life while receiving treatment. In extreme situations, side effects that occur during treatment may prevent you from continuing treatment or from having a planned surgery* DCE attribute text: *Impact on energy levels, daily activities, and quality of life while receiving treatment*	Moderate	Severe Moderate None or mild
**Long-term side effects** Attribute description: *Long-term side effects of treatment continue to cause problems even after treatment is finished. Long-term side effects may include brain fog, nerve damage and pain, shortness of breath, or fatigue. They may also include events such as loss of fertility or early menopause. Long-term side effects can impact your quality of life, energy levels, and your ability to complete day-to-day tasks* DCE attribute text: *X out of 100 (xx%) patients will develop post-treatment, potentially irreversible, side effects that continue to affect quality of life*	50%	95% 50% 0%

Abbreviations: DCE, discrete choice experiment; DFS, disease-free survival; EFS, event-free survival; pCR, pathological complete response; RFS, recurrence-free survival; Notes: * Five-year OS values 40%, 60%, and 80% replaced 25%, 50%, and 95% mid-data collection. Bolded terms indicate the attribute name and italics indicates text used verbatim in the survey.

**Table 2:** Participant clinical and demographic characteristics.

Characteristic	Overall *n* (%)	GI Cancer Patients *n* (%)	Breast Cancer Patients *n* (%)	Lung Cancer Patients *n* (%)
**Current age**	**(** ***n*** ** = 103)**	**(** ***n*** ** = 40)**	**(** ***n*** ** = 32)**	**(** ***n*** ** = 31)**
Mean (SD); median (Min, Max)	58.4 (8.9); 59.0 (43.0, 75.0)	58.1 (8.0); 60.0 (44.0, 75.0)	58.6 (9.5); 59.5 (43.0, 75.0)	58.5 (9.6); 57.0 (44.0, 75.0)
**Type of cancer diagnosed**	**(** ***n*** ** = 103)**	**(** ***n*** ** = 40)**	**(** ***n*** ** = 32)**	**(** ***n*** ** = 31)**
GI cancer	40 (38.8%)	40 (100.0%)	0	0
Breast cancer	32 (31.1%)	0	32 (100.0%)	0
Lung cancer	31 (30.1%)	0	0	31 (100.0%)
**Experienced cancer recurrence**	**(** ***n*** ** = 103)**	**(** ***n*** ** = 40)**	**(** ***n*** ** = 32)**	**(** ***n*** ** = 31)**
No	74 (71.8%)	26 (65.0%)	24 (75.0%)	24 (77.4%)
Yes	29 (28.2%)	14 (35.0%)	8 (25.0%)	7 (22.6%)
**Diagnosed with other primary cancers**	**(** ***n*** ** = 103)**	**(** ***n*** ** = 40)**	**(** ***n*** ** = 32)**	**(** ***n*** ** = 31)**
No	84 (81.6%)	33 (82.5%)	28 (87.5%)	23 (74.2%)
Yes	19 (18.4%)	7 (17.5%)	4 (12.5%)	8 (25.8%)
Genitourinary or gynecologic cancer (e.g., ovarian, cervical, prostate, kidney, bladder)	8 (42.1%)	2 (28.6%)	1 (25.0%)	5 (62.5%)
Head and neck cancer (e.g., salivary gland, nasopharyngeal, throat, lip or oral cavity)	6 (31.6%)	2 (28.6%)	2 (50.0%)	2 (25.0%)
Skin cancer	4 (21.1%)	2 (28.6%)	1 (25.0%)	1 (12.5%)
Breast cancer	1 (5.3%)	1 (14.3%)	0	0
**Had dependent children at time of diagnosis**	**(** ***n*** ** = 102)**	**(** ***n*** ** = 40)**	**(** ***n*** ** = 31)**	**(** ***n*** ** = 31)**
No	99 (97.1%)	40 (100.0%)	29 (93.5%)	30 (96.8%)
Yes	3 (2.9%)	0	2 (6.5%)	1 (3.2%)
**Would have liked to have children at the time of their cancer diagnosis**	**(** ***n*** ** = 103)**	**(** ***n*** ** = 40)**	**(** ***n*** ** = 32)**	**(** ***n*** ** = 31)**
No	103 (100.0%)	40 (100.0%)	32 (100.0%)	31 (100.0%)
**Province or territory**	**(** ***n*** ** = 103)**	**(** ***n*** ** = 40)**	**(** ***n*** ** = 32)**	**(** ***n*** ** = 31)**
West coast (British Columbia)	15 (14.6%)	5 (12.5%)	7 (21.9%)	3 (9.7%)
Prairie provinces (Alberta, Saskatchewan, Manitoba)	27 (26.2%)	10 (25.0%)	5 (15.6%)	12 (38.7%)
Central Canada (Quebec and Ontario)	29 (28.2%)	16 (40.0%)	9 (28.1%)	4 (12.9%)
Atlantic Canada (Nova Scotia, New Brunswick, Newfoundland and Labrador, Prince Edward Island)	23 (22.3%)	7 (17.5%)	7 (21.9%)	9 (29.0%)
Northern territories (Yukon, Nunavut, Northwest Territories)	9 (8.7%)	2 (5.0%)	4 (12.5%)	3 (9.7%)
**Area of residence**	**(** ***n*** ** = 103)**	**(** ***n*** ** = 40)**	**(** ***n*** ** = 32)**	**(** ***n*** ** = 31)**
City	67 (65.0%)	26 (65.0%)	22 (68.8%)	19 (61.3%)
Suburban	13 (12.6%)	4 (10.0%)	3 (9.4%)	6 (19.4%)
Town	5 (4.9%)	1 (2.5%)	4 (12.5%)	0
Rural	18 (17.5%)	9 (22.5%)	3 (9.4%)	6 (19.4%)
**Sex at birth**	**(** ***n*** ** = 103)**	**(** ***n*** ** = 40)**	**(** ***n*** ** = 32)**	**(** ***n*** ** = 31)**
Male	55 (53.4%)	31 (77.5%)	0	24 (77.4%)
Female	48 (46.6%)	9 (22.5%)	32 (100.0%)	7 (22.6%)
**Race/ethnicity**	**(** ***n*** ** = 103)**	**(** ***n*** ** = 40)**	**(** ***n*** ** = 32)**	**(** ***n*** ** = 31)**
White	67 (65.0%)	26 (65.0%)	21 (65.6%)	20 (64.5%)
Black	14 (13.6%)	7 (17.5%)	6 (18.8%)	1 (3.2%)
Asian	17 (16.5%)	5 (12.5%)	4 (12.5%)	8 (25.8%)
Indigenous	3 (2.9%)	0	1 (3.1%)	2 (6.5%)
Latin American	3 (2.9%)	2 (5.0%)	0	1 (3.2%)
**Household income**	**(** ***n*** ** = 103)**	**(** ***n*** ** = 40)**	**(** ***n*** ** = 32)**	**(** ***n*** ** = 31)**
$0–$34,999	10 (9.7%)	5 (12.5%)	3 (9.4%)	2 (6.5%)
$35,000–$69,999	47 (45.6%)	17 (42.5%)	13 (40.6%)	17 (54.8%)
$70,000–$99,999	43 (41.7%)	18 (45.0%)	14 (43.8%)	11 (35.5%)
$100,000–$149,000	3 (2.9%)	0	2 (6.2%)	1 (3.2%)
**Employment status**	**(** ***n*** ** = 103)**	**(** ***n*** ** = 40)**	**(** ***n*** ** = 32)**	**(** ***n*** ** = 31)**
Working, full time (>30 h/week) or part-time (0–30 h/week)	6 (5.8%)	2 (5.0%)	2 (6.2%)	2 (6.4%)
Retired	37 (35.9%)	13 (32.5%)	12 (37.5%)	12 (38.7%)
Stay at home parent	19 (18.4%)	7 (17.5%)	8 (25.0%)	4 (12.9%)
Not working due to cancer	31 (30.1%)	9 (22.5%)	10 (31.2%)	12 (38.7%)
Not working for other reasons	10 (9.7%)	9 (22.5%)	0	1 (3.2%)
**Highest level of education**	**(** ***n*** ** = 103)**	**(** ***n*** ** = 40)**	**(** ***n*** ** = 32)**	**(** ***n*** ** = 31)**
Did not complete high school education	10 (9.7%)	5 (12.5%)	4 (12.5%)	1 (3.2%)
High school diploma or equivalent	40 (38.8%)	14 (35.0%)	11 (34.4%)	15 (48.4%)
Undergraduate degree or college certificate	46 (44.7%)	17 (42.5%)	16 (50.0%)	13 (41.9%)
Post-graduate degree	7 (6.8%)	4 (10.0%)	1 (3.1%)	2 (6.5%)

Abbreviations: GI, gastrointestinal; SD, standard deviation. Notes: Bolded rows indicate a section header; each section pertains to a specific characteristic.

**Table 3:** Participant responses to concluding questions.

Characteristic	Overall *n* (%)	GI Cancer *n* (%)	Breast Cancer *n* (%)	Lung Cancer *n* (%)
**1. Respondent assumed a better alternative endpoint results in a better overall survival**	**(** ***n*** ** = 103)**	**(** ***n*** ** = 40)**	**(** ***n*** ** = 32)**	**(** ***n*** ** = 31)**
No	6 (5.8%)	3 (7.5%)	1 (3.1%)	2 (6.5%)
Yes	97 (94.2%)	37 (92.5%)	31 (96.9%)	29 (93.5%)
**2. Respondent thinks treatments should be available earlier based on alternative endpoint data rather than waiting for overall survival**	**(** ***n*** ** = 103)**	**(** ***n*** ** = 40)**	**(** ***n*** ** = 32)**	**(** ***n*** ** = 31)**
No	57 (55.3%)	22 (55.0%)	19 (59.4%)	16 (51.6%)
Yes	46 (44.7%)	18 (45.0%)	13 (40.6%)	15 (48.4%)
**3. If alternative endpoint data could predict overall survival, respondent would consider a new treatment that reduces disease advancement compared to the standard treatment even if its five-year survival rate is not yet known**	**(** ***n*** ** = 103)**	**(** ***n*** ** = 40)**	**(** ***n*** ** = 32)**	**(** ***n*** ** = 31)**
Would choose the new treatment	44 (42.7%)	18 (45.0%)	13 (40.6%)	13 (41.9%)
Would consider the new treatment	3 (2.9%)	0	2 (6.2%)	1 (3.2%)
Would not choose the new treatment	56 (54.4%)	22 (55.0%)	17 (53.1%)	17 (54.8%)
**4. If alternative endpoint data could not predict overall survival, respondent would consider a new treatment that reduces disease advancement compared to the standard treatment even if its five-year survival rate is not yet known**	**(** ***n*** ** = 103)**	**(** ***n*** ** = 40)**	**(** ***n*** ** = 32)**	**(** ***n*** ** = 31)**
Would choose the new treatment	40 (38.8%)	17 (42.5%)	12 (37.5%)	11 (35.5%)
Would consider the new treatment	6 (5.8%)	1 (2.5%)	2 (6.2%)	3 (9.7%)
Would not choose the new treatment	57 (55.3%)	22 (55.0%)	18 (56.2%)	17 (54.8%)
**5. Respondent would be interested in trying a new treatment with reduced disease advancement if overall survival was similar**	**(** ***n*** ** = 103)**	**(** ***n*** ** = 40)**	**(** ***n*** ** = 32)**	**(** ***n*** ** = 31)**
Would choose the new treatment	34 (33.0%)	15 (37.5%)	7 (21.9%)	12 (38.7%)
Would consider the new treatment	13 (12.6%)	3 (7.5%)	8 (25.0%)	2 (6.5%)
Would not choose the new treatment	56 (54.4%)	22 (55.0%)	17 (53.1%)	17 (54.8%)
**6. If alternative endpoints predicted overall survival, respondent would have answered questions differently**	**(** ***n*** ** = 103)**	**(** ***n*** ** = 40)**	**(** ***n*** ** = 32)**	**(** ***n*** ** = 31)**
No	102 (99.0%)	40 (100.0%)	32 (100.0%)	30 (96.8%)
Yes	1 (1.0%)	0	0	1 (3.2%)
**7. Respondent sought second medical opinion for diagnosis or treatment plan**	**(** ***n*** ** = 103)**	**(** ***n*** ** = 40)**	**(** ***n*** ** = 32)**	**(** ***n*** ** = 31)**
No	23 (22.3%)	5 (12.5%)	10 (31.2%)	8 (25.8%)
Yes	80 (77.7%)	35 (87.5%)	22 (68.8%)	23 (74.2%)
**8. Respondent followed first treatment plan as suggested by initial doctor**	**(** ***n*** ** = 103)**	**(** ***n*** ** = 40)**	**(** ***n*** ** = 32)**	**(** ***n*** ** = 31)**
No	66 (64.1%)	29 (72.5%)	16 (50.0%)	21 (67.7%)
Yes	37 (35.9%)	11 (27.5%)	16 (50.0%)	10 (32.3%)

Abbreviations: GI, gastrointestinal. Notes: Bolded rows indicate a section header; each section pertains to a specific survey question.

## Data Availability

DCE data and all study materials are available from the corresponding author upon reasonable request.

## Supplementary Materials

The following supporting information can be downloaded at: https://www.mdpi.com/article/10.3390/curroncol33060308/s1, Figure S1: Discrete choice experiment: instructions; Figure S2: Comprehension check questions; Figure S3: Additional questions; Table S1: Recruitment targets and explanation; Table S2: Characteristics of participants with GI cancer; Table S3: Characteristics of participants with lung cancer; Table S4: Characteristics of participants with breast cancer; Table S5: Discrete choice experiment—overall results; Table S6: Willingness to trade overall survival in the standard-of-care treatment.

## Abbreviations

The following abbreviations are used in this manuscript:

**Acronym**	**Definition**
CAD	Canadian dollars
DCE	Discrete choice experiment
DFS	Disease-free survival
EFS	Event-free survival
FAS	Full analysis set
FDA	Food and Drug Administration
GI	Gastrointestinal
IQR	Interquartile range
IRB	Independent Review Board
NSCLC	Non-small cell lung cancer
RFS	Recurrence-free survival
SD	Standard deviation
SOC	Standard-of-care
