# Supplementary material for: Evaluating Patient Preferences for Clinical Trial Endpoints in Early-Stage Cancer: A Discrete Choice Experiment in Canada
Source: Curr Oncol. 2026 May 26;33(6):308. doi: 10.3390/curroncol33060308 (PMC13297726; doi:10.3390/curroncol33060308)
Supplement: Supplementary file 1 [file curroncol-33-00308-s001.zip › curroncol-4224612-supplementary.pdf]

Title: Evaluating patient preferences for clinical trial endpoints in early-stage cancer: a discrete choice experiment in Canada

Supplementary tables and figures

Figure S1: Background information on clinical trial endpoints in oncology

Welcome!

Thank you for agreeing to participate in this study.

You have been asked to participate in this study because you have been diagnosed with early-stage breast, gastrointestinal (e.g. liver, stomach, esophageal), or lung cancer. There is a good chance that you already know a lot of the information presented below; however, we ask that you read it carefully anyways. People can have slightly different definitions of terms related to cancer treatment, and we would like all participants of this survey to be thinking about them in the same way. This will allow us to accurately combine and compare all participants' responses.

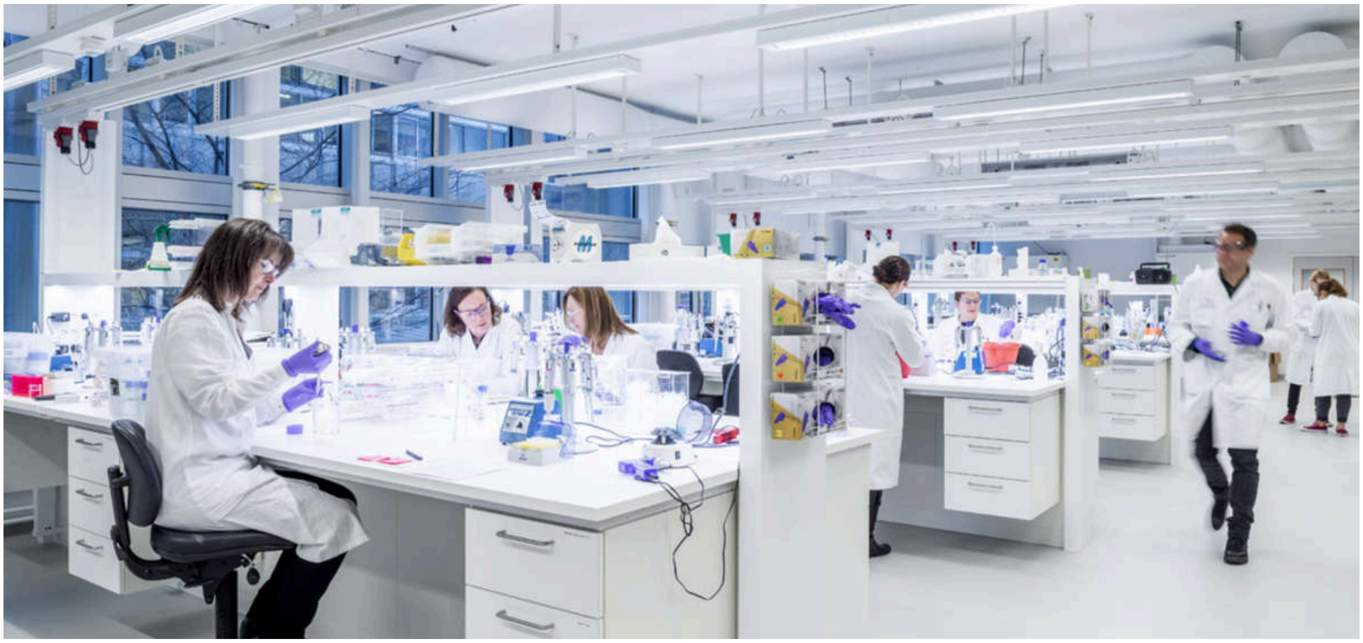

Clinical trials

Clinical trials are studies that involve human participants and aim to evaluate how well an intervention can treat a disease and/or the safety of the intervention. Interventions can include drugs, vaccines, procedures, and more. Results from clinical trials can be used to determine which treatments are best for specific populations and support the approval and public reimbursement of different drugs in Canada. Because these results are only applicable to specific populations, the results from clinical trials in one cancer type may not carry over to another cancer type. For example, a drug may be shown to be

beneficial for colon cancer, but that does not necessarily mean the same drug will be beneficial for breast cancer.

Results from clinical trials that describe treatment success, or efficacy, are called efficacy endpoints. Some commonly used efficacy endpoints are **overall survival**, and alternative endpoints that are time-based measures of **disease advancement** such as disease- or recurrence-free survival, and event-free survival, and **pathological complete response**. These alternative endpoints can be useful in assessing treatment efficacy in early-stage cancers as they can be observed earlier compared to overall survival within a clinical trial.

When evaluating the safety of an intervention, we often talk about **side effects**. Side effects can be **mild, moderate**, or **severe**, and can occur during treatment (**short-term side effects**) or after treatment has been completed (**long-term side effects**).

Efficacy endpoints

Overall survival

Among a group of people who are diagnosed with cancer and undergoing a similar treatment (e.g. those enrolled in a clinical trial), the overall survival describes the percent who are alive, with or without cancer, for a given time (e.g. five years) after starting treatment. It is the most common endpoint used in cancer trials because it captures one goal of treatment: prolonging life.

Often, this is expressed as *'The percent of people alive five years after starting treatment'*. For example, if 80 out of 100 people who start a particular drug to treat their cancer are alive (with or without cancer) after five years, we would say that this drug is associated with *'80% five-year overall survival'*.

Alternative endpoints

There are alternative endpoints that can be used to measure treatment benefit in clinical trials in addition to overall survival. Particularly in early-stage cancers, these endpoints can be observed at earlier time points within a clinical trial period and can capture other goals of cancer treatment: prolonging time spent cancer or event-free and the associated impacts to quality of life. Alternative endpoints, such as recurrence-free, disease-free or event-free survival, can inform on treatment benefit based on its ability to prevent **recurrence**, or an event that the treatment was intended to prevent or delay (e.g., onset of new symptoms).

Time-based measures of disease advancement

Among a group of people who are diagnosed with cancer and undergoing a similar treatment (e.g. those enrolled in a clinical trial), we will define disease advancement as the proportion of patients who have no evidence of:

- an event that the treatment was intended to prevent or delay (e.g., onset of new symptoms, cancer progression) (event-free survival)
- the original cancer returning (recurrence-free survival)
- the original cancer returning or a new kind of cancer (disease-free survival)
- and are alive

For example, if 80 out of 100 people who start a particular drug to treat their cancer are alive without cancer after two years, we would say that this drug is associated with *'80% two-year recurrence-free survival'*.

Pathological complete response (pCR)

Pathological complete response (pCR) is achieved when there are no signs of cancer in a person's tissue samples that are examined after treatment. Tissue samples are removed during surgery or biopsy and are examined under a microscope to see if the treatment was successful.

Relevance of alternative endpoints

Alternative endpoints from time-based measures of **disease advancement** or **pCR** can provide insight into the quality-of-life people might expect after treatment. For example, being 'cancer-free' means a person may not have to undergo further treatment or experience the associated side effects. Avoiding cancer **recurrence** post treatment means they won't experience further cancer symptoms or have to start a new line of treatment.

Endpoints and cancer treatment approval in Canada

Traditionally, cancer drugs have been approved based on their ability to demonstrate **overall survival**. While this is important information, it means that once a new, potentially effective treatment has been developed, it will not be available for use **until sufficient data on overall survival have been measured.**

To decrease the amount of time patients must wait until a potentially curative treatment is available for use, new therapies in early-stage cancers may be approved based on their ability to demonstrate efficacy via the alternative endpoints which can be measured at earlier time points in advance of overall survival (time-based measures of **disease advancement** and **pCR**). It is important to understand, however, that when the treatment is approved based on an alternative endpoint, the overall survival is not yet known.

Side effects

People may experience a wide range of side effects (also referred to as 'adverse events') while undergoing cancer treatment. The side effects of cancer treatment may appear gradually and may last after cancer treatment has been completed.

Usually, **short-term** side effects occur during treatment and go away once treatment is complete. These side effects may include hair loss, nausea, or vomiting. In extreme situations, side effects which occur during treatment may prevent a person from continuing treatment or from having a planned surgery.

Some side effects of treatment are **long-term** and continue to cause problems even after treatment is finished. Long-term side effects may include brain fog, nerve damage and pain, shortness of breath, or fatigue. They may also include events such as loss of fertility or early menopause. Both short term and long-term side effects can impact a person's quality of life, energy levels, and their ability to complete day-to-day tasks.

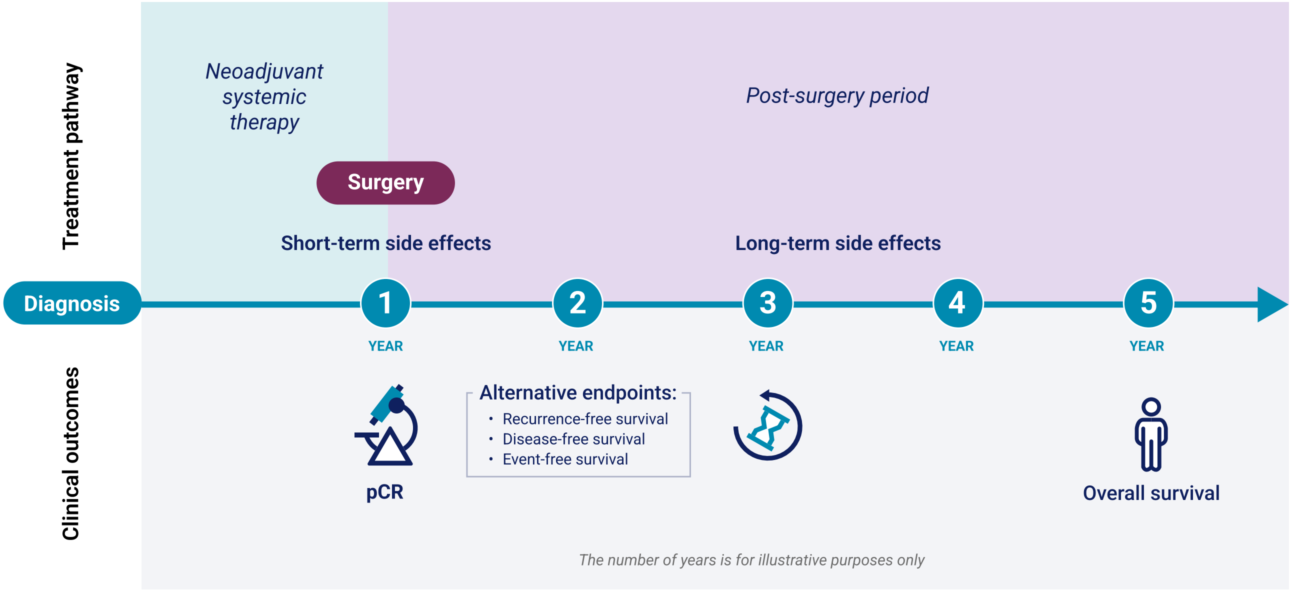

Next

Understanding preferences of Canadians for non-overall survival endpoints in oncology: A discrete choice experiment

Attribute and level descriptions

Overall survival

For this exercise, we assume that overall survival describes the proportion of people who are diagnosed with cancer and are alive, with or without cancer, five years after starting treatment.

Possible values:

Not yet known

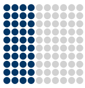

40%
40 out of 100 patients are alive *five* years after treatment

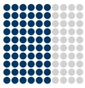

60%
60 out of 100 patients are alive *five* years after treatment

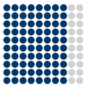

80%
80 out of 100 patients are alive *five* years after treatment

Disease advancement

Among a group of people who are diagnosed with cancer and undergoing a similar treatment (e.g. those enrolled in a clinical trial), the percent who have no evidence of disease advancement (**disease-free survival**, **recurrence-free survival**, **event-free survival**) over a given period (e.g. two years) after starting treatment.

Possible values:

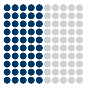

50%
For 50 out of 100 patients, cancer has not progressed *two* years after initiating treatment

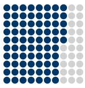

75%
For 75 out of 100 patients, cancer has not progressed *two* years after initiating treatment

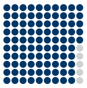

95%
For 95 out of 100 patients, cancer has not progressed *two* years after initiating treatment

Pathological complete response (pCR)

Pathological complete response, or pCR, is achieved when there are no surviving cancer cells in a person's tissue samples removed with surgery. Tissue samples are removed during surgery and are examined under a microscope to see if the treatment was successful.

Possible values:

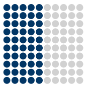

50%
For 50 out of 100 patients, there are no signs of cancer in the tissue samples removed during surgery after completing **systemic therapy**

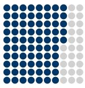

75%
For 75 out of 100 patients, there are no signs of cancer in the tissue samples removed during surgery after completing **systemic therapy**

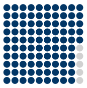

95%
For 95 out of 100 patients, there are no signs of cancer in the tissue samples removed during surgery after completing **systemic therapy**

Short-term side effects

Short-term side effects occur during treatment and go away once treatment is complete. These side effects may include hair loss during chemotherapy, nausea, or vomiting. These side effects can impact energy levels, daily activities, and quality-of-life while receiving treatment. In extreme situations, side effects which occur during treatment may prevent you from continuing treatment or from having a planned surgery.

Possible values:

None or mild

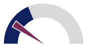

Impact to energy levels, daily activities, and quality-of-life while receiving treatment

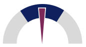

Moderate
Impact to energy levels, daily activities, and quality-of-life while receiving treatment

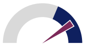

Severe
Impact to energy levels, daily activities, and quality-of-life while receiving treatment

Long-term side effects

Long-term side effects of treatment continue to cause problems even after treatment is finished. Long-term side effects may include brain fog, nerve damage and pain, shortness of breath, or fatigue. They may also include events such as loss of fertility or early menopause. Long-term side effects can impact your quality-of-life, energy levels, and your ability to complete day-to-day tasks.

Possible values:

0%
0 out of 100 patients will develop post-treatment, potentially irreversible, side effects that continue to affect quality-of-life

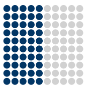

50%
50 out of 100 patients will develop post-treatment, potentially irreversible, side effects that continue to affect quality-of-life

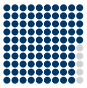

95%
95 out of 100 patients will develop post-treatment, potentially irreversible, side effects that continue to affect quality-of-life

Next

Figure S2: DCE instructions

6/19/25, 12:14 PM Endpoints DCE

Understanding preferences of Canadians for non-overall survival endpoints in oncology: A discrete choice experiment

Access to introduction pages

CONSENT (2) INTRODUCTION (3) COMPREHENSIO (4) DCE (5) CONCLUDING QUESTIONS (6) BASELINE QUESTIONNAIRE

DCE completion instructions

For the purposes of completing the next ten choice tasks, we will ask that you please imagine you have an early-stage cancer that can be treated with the current standard of care (e.g., systemic therapy followed by surgery). This may be different to your own cancer experience.

The treatment currently available for your cancer provides a **60%** *chance of surviving five years*. There is a **new treatment** available; however, five-year overall survival with this treatment is **not yet known** as the study may be ongoing and may take longer to collect this data.

Please note that the frequency (how often) and duration (for how long) you take the treatment is *the same* for both the current standard of care treatment and the **new treatment**.

For each question (1 per page) please indicate, based on the treatment characteristics described, which option you prefer between the standard of care treatment and the **new treatment** to treat your cancer.

Please think carefully about which outcomes are most important to you and make your decisions thoughtfully, considering all the features of each choice. For some questions, it may be difficult to make a choice. Please choose the option you prefer the most as best you can – there are no wrong answers.

Example of DCE choice set

Figure S3: Additional questions

Understanding preferences of Canadians for non-overall survival endpoints in oncology: A discrete choice experiment

Access to introduction pages

CONSENT INTRODUCTION 3 COMPREHENSION DCE CONCLUDI G QUESTIONS BASELINE QUESTION A RE

Comprehension check questions

1. Which of the following is not true about **short-term side effects:** *(select the incorrect statement)*

   If severe, they may prevent you from continuing treatment or from having a planned surgery

   Are likely to cause nerve damage and pain for the rest of your life

   Can impact your quality of life, energy levels, and your ability to complete day-to-day tasks

2. **Alternative endpoints** such as time-based measures of disease advancement measured two years after beginning treatment for a group of patients in a clinical trial can tell us: *(select the correct statement)*

   How many people dropped out of the clinical trial

   How many people's disease progressed (e.g., had a relapse or recurrence)

   How many people are likely to survive until five years after starting treatment

3. **Long-term side effects:** *(select the correct statement)*

   Occur during treatment, and resolve once you stop taking the treatment

   There are no long-term side effects associated with cancer treatment

   Occur even after completing treatment and may never resolve

Time to treatment approval and predictive ability of alternative endpoints questions

Treatments that are approved based on alternative-endpoint data (e.g., reduced side-effects, better pCR results, or not experiencing disease advancement) will be available to patients earlier than if decision-makers waited for five-year overall survival data to become available.

If a new treatment performs better than the standard-of-care on an alternative endpoint (e.g. disease advancement outcomes), the time lived cancer-free is expected to be longer with the new treatment than with the standard-of-care treatment.

There is some evidence that alternative endpoint data can predict overall survival (e.g., fewer people experiencing disease advancement at 2 years meant better overall survival at 5 years, or more people experiencing disease advancement at 2 years meant worse overall survival at 5 years); however, not enough information is available to confirm if this is true or not for all kinds of endpoints.

1. In the above questions, when overall survival was unknown for a treatment, did you make the assumption that a better alternative endpoint, (e.g. **disease advancement** or **pCR**), would result in a better overall survival?

- Yes
- No
- Unsure
- I don't understand the question

2. Do you think treatments should be available earlier, based on alternative endpoint data, rather than waiting until overall survival is known?

- Yes
- No
- Unsure
- I don't understand the question

3. If researchers proved that alternative endpoint data **COULD** reliably predict overall survival, would you consider trying a new treatment that reduces disease advancement compared to the standard treatment, even if its five-year survival rate is not yet known?

- I would **not choose the new treatment** until overall survival was known
- I would **consider taking the new treatment** based on better performance on alternative endpoints, but am not quite sure what I would choose
- I would **choose the new treatment** based on better performance on alternative endpoints, before five-year overall survival was known
- I **don't understand** the question

4. If researchers proved that alternative endpoint data **COULD NOT** reliably predict overall survival, would you consider trying a new treatment that reduces disease advancement compared to the standard treatment, even if its five-year survival rate is not yet known?

- I would **not choose the new treatment** until overall survival was known
- I would **consider taking the new treatment** based on better performance on alternative endpoints, but am not quite sure what I would choose
- I would **choose the new treatment** based on better performance on alternative endpoints, before five-year overall survival was known
- I **don't understand** the question

5. If you were to choose between two treatments where five-year overall survival was about the same, and the new treatment reduced disease advancement, would you be interested in trying the new treatment?

- I would **not choose** the new treatment
- I would **consider taking the new treatment**
- I would **choose the new treatment**
- I **don't understand** the question

6. If you had been told the alternative endpoints presented in the choice tasks (i.e. **disease advancement** or **pCR**) strongly predicted five-year overall survival, would you have answered any of the questions differently?

- Yes
- No
- Unsure
- I don't understand the question

7. When you received your cancer diagnosis, did you seek a second medical opinion for your diagnosis or treatment plan?

- Yes
- No

8. Did you follow the first treatment plan as suggested by your initial doctor?

- Yes
- No

Next

Supplemental Table S1: Recruitment targets and explanation

Sampling Target	n (%)	Explanation
**Cancer Type**		
Breast	30 (30%)	
Lung	30 (30%)	Recruiting 30% each of breast, lung and GI cancer was consistent with the approximate population distributions.[1] Within the GI group, the goal was to include 10% each with liver, gastric, and esophageal cancers (n=10 each), to ensure representation of less common but clinically relevant GI cancers.[1] If this distribution was not feasible, the remaining GI participants were to be drawn from other GI cancer types, such as colorectal or pancreatic cancer.
GI	30 (30%)<br>See below for breakdown:	
Liver	≥10 (10%)	
Gastric	≥10 (10%)	
Esophageal	≥10 (10%)	
Other GI	No target	
Any of the above	10 (10%)	
**Age at diagnosis**		
<50 years old at time of diagnosis	≥15 (15%)<br>≥5 (5%) each cancer group	These targets reflected the epidemiology of each cancer type in Canada; for example, 83% of breast cancers and 98% of lung cancers are diagnosed in people over 50,[2] while the highest incidence of liver and esophageal cancer occurs in people aged 70 and older.[3,4]
>70 years old at time of diagnosis	≥5 (5%)<br>≥2 (2%) each cancer group	
**Racial/ethnic minorities**		
South Asian	≥5 (5%)	These proportions were based on population estimates indicating that 7% of Canadians identify as South Asian, and 5% as Black or Southeast/East Asian.[5]
Black	≥5 (5%)	
Southeast Asian/East Asian	≥5 (5%)	
**Time since diagnosis / treatment**		

Sampling Target	n (%)	Explanation
Currently receiving treatment	≥15 (15%)	These targets acknowledged that newly diagnosed individuals may prioritize treatment outcomes differently from long-term survivors, and that the five-year post-treatment mark is commonly viewed as a clinical milestone indicative of cure.[6]
Diagnosed / treated >5 years ago	≥15 (15%)	
**Relapse experience**		
Experienced at least one relapse of breast, lung, or liver cancer diagnosis	≥25 (25%)	Recurrence rates vary widely by cancer type ranging from 7% to 80% within five years;[7-9] therefore, this criterion was set to ensure representation of experiences across the treatment spectrum.
**Urban vs rural dwellers**		
Reside in a rural location	≥15 (15%)	Recruitment from rural or remote areas, consistent with the estimated 18% of Canadians living in non-urban settings.[10] Participants were categorized as rural if they self-reported living in a rural or remote area; those reporting residence in a city, town, or suburb were considered urban.
**Socioeconomic status**		
Less than a high school education	≥7 (≥7%)	Socioeconomic diversity was addressed by targeting at least 7% of participants with less than a high school education, reflecting the proportion of Canadian adults aged 25–64 with below upper secondary education.[11]
Low income (<$35,000 CAD net household income)	≥7 (≥7%)	Additionally, at least 7% of the sample was to report a household income below $35,000 CAD after taxes, based on Canadian definitions of low income as 50% below the national median ($68,400 CAD in 2021).[12,13]

Abbreviations: CAD, Canadian dollar; GI, gastrointestinal.

Supplemental Table S2: Characteristics of participants with GI cancer

Characteristic	GI cancer patients, n(%)
**Type of GI cancer**	**(n = 40)**
Hepatocellular carcinoma	12 (30.0%)
Esophageal	11 (27.5%)
Gastric	9 (22.5%)
Cholangiocarcinoma	8 (20.0%)
**Stage at diagnosis**	**(n = 40)**
Stage 0	11 (27.5%)
Stage 1	22 (55.0%)
Stage A	7 (17.5%)
**Diagnosis year**	**(n = 40)**
2014-2019	10 (25.0%)
2020-2025	30 (75.0%)
**Treatment start year**	**(n = 40)**
2014-2019	10 (25.0%)
2020-2025	30 (75.0%)
**Treatment status**	**(n = 40)**
Currently receiving treatment	5 (12.5%)
Restarted treatment for lung cancer after disease recurrence or progression	13 (32.5%)
Completed or terminated treatment within the last 5 years	20 (50.0%)
Completed or terminated treatment more than 5 years ago	2 (5.0%)
**Treatment completion year**	**(n = 35)**
2014-2019	5 (14.3%)
2020-2025	30 (85.7%)
**Types of treatment**	**(n = 40)**
Surgery	40 (100.0%)
Number of surgeries	**(n = 40)**

Characteristic	GI cancer patients, n(%)
1	40 (100.0%)
Chemoradiotherapy	35 (87.5%)
Radiation	1 (2.5%)
Number of weeks of radiation/chemoradiation treatment	**(n = 36)**
5	2 (5.6%)
6	13 (36.1%)
7	15 (41.7%)
8	4 (11.1%)
9	2 (5.6%)
Ablation therapy	9 (22.5%)
Frequency	**(n = 9)**
Multiple times a week (e.g., daily or almost daily)	1 (11.1%)
Once a week	1 (11.1%)
Once a month (e.g., 3- or 4- week treatment cycles)	1 (11.1%)
Twice a month (e.g., every other week)	6 (66.7%)
Chemotherapy	2 (5.0%)
Frequency	**(n = 2)**
Once a month (e.g., 3- or 4- week treatment cycles)	2 (100.0%)
Other	1 (2.5%)
Frequency	**(n = 1)**
Once a week	1 (100.0%)
**Total treatment time (years)**	**(n = 40)**
Mean (SD); Median (Min, Max)	2.2 (0.6); 2.1 (1.0, 3.5)
**Side effects during treatment**	**(n = 40)**
Mild side effects not affecting quality of life	9 (22.5%)

Characteristic	GI cancer patients, n(%)
Moderate side effects impacting quality of life	31 (77.5%)
**Side effects since treatment**	**(n = 22)**
Still has mild side effects that don't affect quality of life	3 (13.6%)
Temporary side effects (longer than a month) after finishing treatment, currently resolved and not affecting quality of life	14 (63.6%)
Temporary side effects (within a month) after finishing treatment, currently resolved and not affecting quality of life	5 (22.7%)

Abbreviations: GI, gastrointestinal; SD, standard deviation.

Supplemental Table S3: Characteristics of participants with lung cancer

Characteristic	Lung cancer patients, n(%)
**Type of lung cancer**	**(n = 31)**
Non-small cell	24 (77.4%)
Stage 0 (carcinoma in situ or adenocarcinoma in situ)	4 (16.7%)
Stage I	18 (75.0%)
Stage II	2 (8.3%)
Small cell	7 (22.6%)
**Mutations tested positive for**	**(n = 24)**
EGFR	24 (100.0%)
ALK	1 (4.2%)
**Diagnosis year**	**(n = 31)**
2013-2019	4 (12.9%)

Characteristic	Lung cancer patients, n(%)
2020-2025	27 (87.1%)
**Treatment start year**	**(n = 31)**
2013-2019	4 (12.9%)
2020-2025	27 (87.1%)
**Treatment status**	**(n = 31)**
Currently receiving treatment	5 (16.1%)
Completed or terminated treatment within the last 5 years	19 (61.3%)
Restarted treatment for lung cancer after disease recurrence or progression	7 (22.6%)
**Treatment completion year**	**(n = 26)**
2015-2019	3 (11.5%)
2020-2025	23 (88.5%)
**Types of treatment**	**(n = 31)**
Chemoradiotherapy	20 (64.5%)
Radiation	5 (16.1%)
Number of weeks of radiation/chemoradiation treatment	**(n = 25)**
6	8 (32.0%)
7	14 (56.0%)
8	3 (12.0%)
Surgery	31 (100.0%)
Number of surgeries	**(n = 31)**
1	30 (96.8%)
2	1 (3.2%)
Chemotherapy	5 (16.1%)
Frequency	**(n = 6)**
Once a week	2 (33.3%)
Twice a month (e.g., every other week)	2 (33.3%)

Characteristic	Lung cancer patients, n(%)
Once a month (e.g., 3- or 4- week treatment cycles)	2 (33.3%)
Other	5 (16.1%)
Other treatment for lung cancer frequency	**(n = 5)**
Once a week	1 (20.0%)
Once a month (e.g., 3- or 4- week treatment cycles)	1 (20.0%)
Twice a month (e.g., every other week)	3 (60.0%)
**Total treatment time (years)**	**(n = 31)**
Mean (SD); Median (Min, Max)	2.1 (0.4); 2.1 (0.8, 3.0)
**Side effects during treatment**	**(n = 31)**
Moderate side effects impacting quality of life	24 (77.4%)
Mild side effects not affecting quality of life	7 (22.6%)
**Side effects since treatment**	**(n = 19)**
Still has mild side effects that don't affect quality of life	1 (5.3%)
Temporary side effects (longer than a month) after finishing treatment, currently resolved and not affecting quality of life	13 (68.4%)
Temporary side effects (within a month) after finishing treatment, currently resolved and not affecting quality of life	5 (26.3%)

Abbreviations: ALK, anaplastic lymphoma kinase; SD, standard deviation; EGFR, epidermal growth factor receptor.

Supplemental Table S4: Characteristics of participants with breast cancer

Characteristic	Breast cancer patients, n(%)
**Type of breast cancer**	**(n = 32)**

Characteristic	Breast cancer patients, n(%)
Ductal carcinoma	28 (87.5%)
Lobular carcinoma	4 (12.5%)
**Received positive HER2 test, breast cancer**	**(n = 32)**
Not sure	13 (40.6%)
Yes - estrogen positive	13 (40.6%)
Yes - progesterone positive	8 (25.0%)
**Stage at diagnosis**	**(n = 32)**
Ductal carcinoma in situ (DCIS)	5 (15.6%)
Stage I	26 (81.2%)
Stage II	1 (3.1%)
**Diagnosis year**	**(n = 32)**
2014-2019	9 (28.1%)
2020-2025	23 (71.9%)
**Treatment start year**	**(n = 32)**
2014-2019	9 (28.1%)
2020-2025	23 (71.9%)
**Treatment status**	**(n = 32)**
Currently receiving treatment	6 (18.8%)
Restarted treatment for my breast cancer after disease recurrence or progression	7 (21.9%)
Completed or terminated treatment more than 5 years ago	6 (18.8%)
Completed or terminated treatment within the last 5 years	13 (40.6%)
**Treatment completion year**	**(n = 26)**
2014-2019	7 (26.9%)
2020-2025	19 (73.1%)
**Types of treatment**	**(n = 32)**
Surgery	32 (100.0%)

Characteristic	Breast cancer patients, n(%)
Number of surgeries	**(n = 32)**
1	31 (96.9%)
2	1 (3.1%)
Chemotherapy	30 (93.8%)
Frequency	**(n = 30)**
Multiple times a week (e.g., daily or almost daily)	1 (3.3%)
Twice a month (e.g., every other week)	29 (96.7%)
Radiation	10 (31.2%)
Number of weeks of treatment	**(n = 10)**
5	2 (20.0%)
6	4 (40.0%)
7	3 (30.0%)
8	1 (10.0%)
Other	1 (3.1%)
Frequency	**(n = 1)**
Once a month (e.g., 3- or 4- week treatment cycles)	1 (100.0%)
**Total treatment time (years)**	**(n = 32)**
Mean (SD); Median (Min, Max)	2.2 (0.6); 2.1 (0.7, 3.5)
**Hormonal therapy part of post-operative treatment plan**	**(n = 32)**
Yes (completed)	25 (78.1%)
Yes (receiving)	2 (6.2%)
No	5 (15.6%)
**Years of past hormonal therapy breast cancer treatment**	**(n = 25)**
2	12 (48.0%)
1	11 (44.0%)
Unsure	2 (8.0%)

Characteristic	Breast cancer patients, n(%)
**Years of current/future hormonal therapy breast cancer treatment**	**(n = 2)**
1	2 (100.0%)
**Side effects during treatment**	**(n = 32)**
Mild side effects not affecting quality of life	3 (9.4%)
Moderate side effects impacting quality of life	28 (87.5%)
Severe side effects impacting quality of life	1 (3.1%)
**Side effects after treatment**	**(n = 19)**
Temporary side effects (longer than a month) after finishing treatment, currently resolved and not affecting quality of life	17 (89.5%)
Temporary side effects (within a month) after finishing treatment, currently resolved and not affecting quality of life	2 (10.5%)

Abbreviations: DCIS, Ductal carcinoma in situ; HER2, human epidermal growth factor receptor 2; SD, standard deviation

Supplemental Table 5: Discrete choice experiment – overall results

Attribute	OR (95% CI)	p-value
**Five-year OS**<br>Per 25% fewer survived (with SOC)	3.49 (4.31, 2.82)	<0.01
**Two-year disease advancement**<br>Per 25% fewer advanced (with new treatment)	1.55 (1.26, 1.91)	<0.01
**pCR**<br>Per 25% improvement (with new treatment)	1.20 (0.92, 1.57)	0.17
**Mild/no short-term side effects**<br>Ref: moderate (with new treatment)	1.04 (0.73, 1.49)	0.81
**Mild/no short-term side effects**<br>Ref: severe (with new treatment)	6.67 (4.35, 10.00)	<0.01
**Long-term side effects**<br>Per 25% decrease in risk	1.11 (1.00, 1.23)	<0.05

Abbreviations: CI, confidence interval; OR, odds ratio; OS, overall survival; pCR, pathological complete response; ref, reference; SoC, standard of care

Supplemental Table 6: Willingness to trade overall survival in the standard of care treatment

Attribute	Difference in 5-year SoC OS required to make up for the change in attribute in the new treatment
Severe short-term side effects (Ref: Mild/none)	38.4%
Two-year disease advancement (Per 25% increased risk)	8.9%

pCR (Per 25% less chance of achieving)	3.7%
Long-term side effects (Per 25% increase in risk)	2.1%
Moderate short-term side effects (Ref: Mild/none)	0.8%

Abbreviations: OS, overall survival; pCR, pathological complete response; ref, reference; SoC, standard of care

1. Brenner, D.R.; Gillis, J.; Demers, A.A.; Ellison, L.F.; Billette, J.M.; Zhang, S.X.; Liu, J.L.; Woods, R.R.; Finley, C.; Fitzgerald, N.; et al. Projected estimates of cancer in Canada in 2024. CMAJ : Canadian Medical Association journal = journal de l'Association medicale canadienne 2024, 196, E615-e623, doi:10.1503/cmaj.240095.
2. Canadian Cancer Statistics Advisory Committee. Canadian Cancer Statistics 2021; Canadian Cancer Society: Toronto, ON, 2021.
3. De, P.; Dryer, D.; Otterstatter, M.C.; Semenciw, R. Canadian trends in liver cancer: a brief clinical and epidemiologic overview. Current oncology (Toronto, Ont.) 2013, 20, e40-43, doi:10.3747/co.20.1190.
4. Otterstatter, M.C.; Brierley, J.D.; De, P.; Ellison, L.F.; MacIntyre, M.; Marrett, L.D.; Semenciw, R.; Weir, H.K.; Committee, f.t.C.C.S.S. Esophageal Cancer in Canada: Trends according to Morphology and Anatomical Location. Canadian Journal of Gastroenterology and Hepatology 2012, 26, 649108, doi:https://doi.org/10.1155/2012/649108.
5. Statistics Canada. The Canadian census: A rich portrait of the country's religious and ethnocultural diversity. Available online: https://www150.statcan.gc.ca/n1/daily-quotidien/221026/dq221026b-eng.htm (accessed on May 8 2025).
6. National Cancer Institute. Understanding Cancer Prognosis. Available online: https://www.cancer.gov/about-cancer/diagnosis-staging/prognosis#:~:text=If%20you%20remain%20in%20complete,first%205%20years%20after%20treatment. (accessed on May 8 2024).
7. Uramoto, H.; Tanaka, F. Recurrence after surgery in patients with NSCLC. Translational lung cancer research 2014, 3, 242-249, doi:10.3978/j.issn.2218-6751.2013.12.05.
8. Komen, S.G. Breast Cancer Recurrence. Available online: https://ww5.komen.org/BreastCancer/ReturnofCancerafterTreatment.html (accessed on May 8 2024).
9. Saito, A.; Toyoda, H.; Kobayashi, M.; Koiwa, Y.; Fujii, H.; Fujita, K.; Maeda, A.; Kaneoka, Y.; Hazama, S.; Nagano, H.; et al. Prediction of early recurrence of hepatocellular carcinoma after resection using digital pathology images assessed by machine learning. Modern pathology : an official journal of the United States and Canadian Academy of Pathology, Inc 2021, 34, 417-425, doi:10.1038/s41379-020-00671-z.
10. Statistics Canada. Population of urban areas growing faster than rural areas, although rural population still increasing. Available online: https://www150.statcan.gc.ca/n1/daily-quotidien/220209/g-a003-eng.htm (accessed on August 6 2024). www150.statcan.gc.ca/n1/daily-quotidien/230502/dq230502a-eng.htm (accessed on August 6 2024).

11. Statistics Canada. Educational attainment of the population aged 25 to 64, by age group and sex, Organisation for Economic Co-operation and Development (OECD), Canada, provinces and territories. Available online: https://www150.statcan.gc.ca/t1/tbl1/en/cv.action?pid=3710013001 (accessed on August 6 2024).

12. Statistics Canada. Towards a Poverty Reduction Strategy – A backgrounder on poverty in Canada. Available online: https://www.canada.ca/en/employment-social-development/programs/poverty-reduction/backgrounder.html#h2.1 (accessed on August 6 2024).

13. Statistics Canada. Canadian Income Survey, 2021. Available online: https://www150.statcan.gc.ca/n1/daily-quotidien/230502/dq230502a-eng.htm (accessed on August 6 2024).
